# Beta-hydroxybutyrate, an endogenic NLRP3 inflammasome inhibitor, attenuates stress-induced behavioral and inflammatory responses

**DOI:** 10.1038/s41598-017-08055-1

**Published:** 2017-08-09

**Authors:** Takehiko Yamanashi, Masaaki Iwata, Naho Kamiya, Kyohei Tsunetomi, Naofumi Kajitani, Nodoka Wada, Takahiro Iitsuka, Takahira Yamauchi, Akihiko Miura, Shenghong Pu, Yukihiko Shirayama, Ken Watanabe, Ronald S. Duman, Koichi Kaneko

**Affiliations:** 1 Department of Neuropsychiatry, Faculty of Medicine, Tottori University, Yonago, Japan; 2 Department of Psychiatry, Nara Medical University School of Medicine, Kashihara, Japan; 3 Department of Psychiatry, Teikyo University Chiba Medical Center, Ichihara, Japan; 4 Watanabe Hospital, Tottori, Japan; 5 Departments of Psychiatry and Neurobiology, Yale University School of Medicine, New Haven, Connecticut USA

## Abstract

Neuro-inflammation has been shown to play a critical role in the development of depression. Beta-hydroxybutyrate (BHB) is a ketone body and has recently been reported to exert anti-inflammatory effects via inhibition of NLRP3 inflammasome. Here, we investigated the potential antidepressant and anti-inflammatory effects of BHB on rats exposed to acute and chronic stress. We examined the influence of repeated BHB administration on depressive and anxiety behaviors in a rodent model of chronic unpredictable stress (CUS). Additionally, the influence of acute immobilization (IMM) stress and single BHB administration on hippocampal interleukin-1β (IL-1β) and tumor necrosis factor-α (TNF-α) were assessed. Repeated administration of BHB attenuated CUS-induced depressive- and anxiety-related behaviors. IMM stress increased levels of IL-1β in the hippocampus, while a single pre-administration of BHB attenuated this increase. Although no effect was observed on hippocampal TNF-α levels after 1 h of IMM stress, a single BHB pre-administration reduced hippocampal TNF-α. Our previous report showed that the release of IL-1β and TNF-α caused by stress is tightly regulated by NLRP3 inflammasome. These findings demonstrate that BHB exerts antidepressant-like effects, possibly by inhibiting NLRP3-induced neuro-inflammation in the hippocampus, and that BHB may be a novel therapeutic candidate for the treatment of stress-related mood disorders.

## Introduction

Major depressive disorder (MDD) is characterized by depressed mood, anhedonia, low self-esteem, loss of motivation, sleep disruption, loss of appetite, and other cognitive symptoms^1^. Approximately 33% of patients with depression exhibit little or no improvement when treated with existing typical antidepressants, which commonly act on the monoaminergic systems^2^. Therefore, there is a need to develop novel treatments for depression.

Recent evidence has suggested that pro-inflammatory cytokines play a crucial role in the pathophysiology of MDD^3^, and that blood levels of pro-inflammatory cytokines, such as interleukin-1β (IL-1β), interleukin-6 (IL-6), and tumor necrosis factor-α (TNF-α), are significantly higher in patients with MDD^3, 4^. Preclinical studies have revealed that both stress and IL-1β decrease neurogenesis in the adult hippocampus, and that this effect is associated with the development of depressive behaviors^5^. Moreover, inhibition of IL-1β by administration of IL-1β receptor antagonist or IL-1β receptor deletion abolishes both the anti-neurogenic and depressive behavioral effects of stress^5–7^.

Recently, we demonstrated that immobilization (IMM) stress elevates extracellular IL-1β levels in the adult hippocampus, and that antagonism of the purinergic type 2 X7 receptor (P2X7R), which is the upstream target of the IL-1β production cascade, inhibits the increase in IL-1β levels in the hippocampus, and reverses depressive behaviors induced by chronic unpredictable stress (CUS)^8^. We also revealed that peripheral administration of IL-1β neutralizing antibody blocks depressive-like behaviors induced by CUS^8^. These findings indicate that IL-1β plays a critical role in the development of depression, and that inhibition of IL-1β may represent a novel therapeutic strategy for the treatment of depression^9^.

It is well known that activated caspase-1 is responsible for the maturation and release of IL-1β. Pro-caspase-1 undergoes cleavage to activated caspase-1 via the nucleotide-binding, leucine-rich repeat, pyrin-domain-containing 3 (NLRP3) inflammasome, which is composed of NLRP3, adapter protein apoptosis-associated speck-like protein containing a CARD (ASC), and pro-caspase-1^10^. NLRP3 is therefore a critical factor in the development of neuro-inflammation^11, 12^. Previously, we demonstrated that IMM stress increases the levels of the hippocampal NLRP3 inflammasome^8^, suggesting a role in the pathophysiology of depression.

Recent reports have indicated that beta-hydroxybutyrate (BHB), a ketone body that supports mammalian cell metabolism during states of energy deficits, such as fasting or exercise^13, 14^, reduces NLRP3 inflammasome-mediated production of IL-1β^15^. Research has further revealed that BHB plays a neuroprotective function by exerting anti-oxidative effects and improving mitochondrial function^16–18^. Together, these studies support the hypothesis that BHB may produce antidepressant effects via inhibition of NLRP3 and stress-induced neuro-inflammation.

In the present study, we used a CUS model of depression and IMM stress to evaluate the potential antidepressant and anti-inflammatory effects of BHB. Our results indicate that BHB attenuates stress-induced depressive-like behaviors as well as the elevation of IL-1β and TNF-α in the rodent hippocampus.

## Results

### Subcutaneous single administration of BHB slightly increases BHB levels in the hippocampus

**Figure 1:**
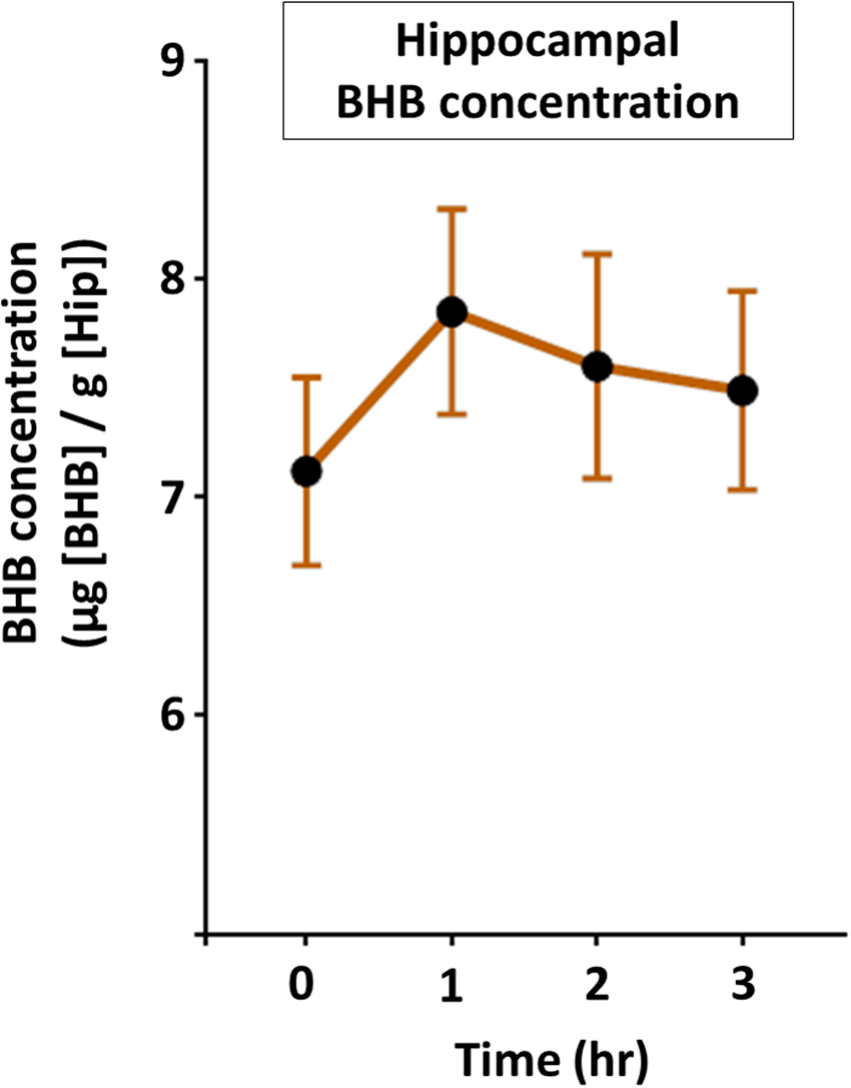
Elevated levels of hippocampal BHB after subcutaneous administration of BHB. Levels of hippocampal BHB measured after subcutaneous injection of BHB are shown (n = 4). The concentration of BHB increased at 1 h after administration, after which gradual decreases in hippocampal BHB levels were observed. BHB: beta-hydroxybutyrate, Hip: hippocampus.

We assessed the concentration of BHB in the hippocampus at 0, 1, 2, and 3 h after subcutaneous single administration of BHB. One hour after injection, we observed an increase in BHB levels relative to baseline. Subsequently, BHB levels in the hippocampus gradually decreased (Fig. 1).

### Depressive- and anxiety-like behavioral effects of CUS are attenuated by BHB administration

**Figure 2:**
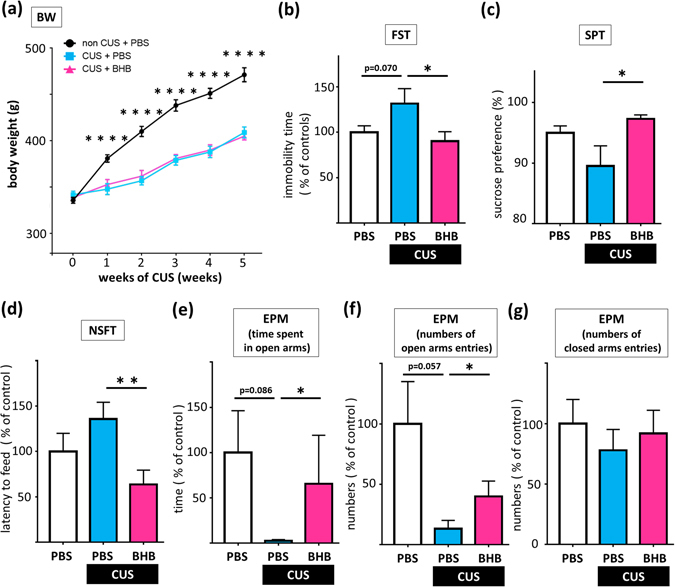
BHB treatment blocks chronic unpredictable stress-induced depressive-like behaviors. (**a**) The BWs of the rats during the CUS procedure are shown. CUS rats exhibited a decreased rate of BW gain at 1 week after starting the CUS procedure, as compared to the non-CUS rats. (**b**) Time spent immobile during the FST is shown. CUS rats showed a trend toward more immobility time relative to rats of the non-CUS control group. BHB significantly decreased the duration of immobility in CUS model rats. (**c**) The results of the SPT are shown. BHB significantly increased the preference for sucrose. (**d**) Latency to feed in the NSFT is shown. BHB significantly reduced the latency to feed in CUS rats. (**e**) Time spent in the open arms in the EPM is shown. CUS tended to reduce time spent in the open arms. BHB significantly attenuated this effect. (**f**) Numbers of open-arm entries in the EPM are shown. CUS tended to decrease the number of open-arm entries, although BHB administration significantly attenuated this effect. (**g**) Numbers of closed-arm entries in the EPM are shown. There was no statistically significant difference. CUS: chronic unpredictable stress, PBS: phosphate buffered saline, BHB: beta-hydroxybutyrate, BW: body weight, FST: forced swim test, SPT: sucrose preference test, NSFT: novelty suppressed feeding test, EPM: elevated plus maze test. *p < 0.05, **p < 0.01, ****p < 0.0001, Error bars: standard error of the mean (SEM).

We examined the influence of CUS on body weight (BW). At the beginning of the experiment, there were no significant differences in BW among the three groups. CUS rats exhibited a decrease in BW gain after the second week of the CUS procedure as compared with non-CUS rats, and the reduced BW gain was not affected by treatment with BHB (F2.45 = 29.522, p < 0.0001, n = 16) (Fig. 2a). We also tested behavioral responses, and found that CUS exposure increased depressive and anxiety-like behaviors measured in the forced swim test (FST), sucrose preference test (SPT), novelty suppressed feeding test (NSFT), and elevated plus maze (EPM), and that chronic BHB administration attenuated these CUS-induced behaviors. In the FST (F2.45 = 3.242, p = 0.048, n = 16), CUS rats showed a trend toward more time spent immobile over the 6-min test period than rats of the non-CUS control group (p = 0.070). Furthermore, BHB administration significantly attenuated CUS-induced increases in time spent immobilized (p = 0.018) (Fig. 2b). In the SPT (X2 = 6.484, p = 0.039, n = 14–16), CUS tended to reduce the preference for sucrose (although not statistically significantly), while BHB attenuated this reduction significantly (p = 0.011) (Fig. 2c). In the NSFT (X2 = 8.892, p = 0.012, n = 16), we observed a trend for increased latency to feed in the CUS model rats (although not statistically significantly), while BHB significantly reduced the latency to feed (p = 0.002) (Fig. 2d). Time spent in the open arms in the EPM (X2 = 6.139, p = 0.046, n = 16) showed that CUS tended to reduce time spent in the open arms (p = 0.086), and this reduction was attenuated by BHB administration (p = 0.012) (Fig. 2e). The number of open-arm entries in the EPM (X2 = 6.273, p = 0.043, n = 16) also tended to be decreased in the CUS relative to the non-CUS group (p = 0.057). BHB significantly attenuated this effect (p = 0.014) (Fig. 2f). There were no statistically significant differences among the groups in terms of the number of closed-arm entries (F2.45 = 0.348, p = 0.708, n = 16) (Fig. 2g). In summary, these results indicated that CUS induces depressive and anxiety-like behavior, and that BHB attenuated these effects.

To know how BHB itself affects the behavior, we evaluated behaviors (FST, SPT, NSFT, and EPM) after 3 weeks BHB administration without the CUS paradigm as a preliminary analysis. BHB alone did not change or improve behaviors in the four behavioral tests relative to the control group (Supplementary Figure 1a–f).

### Regulation of IL-1β, TNF-α, and IL-10 in the hippocampus by stress and BHB

**Figure 3:**
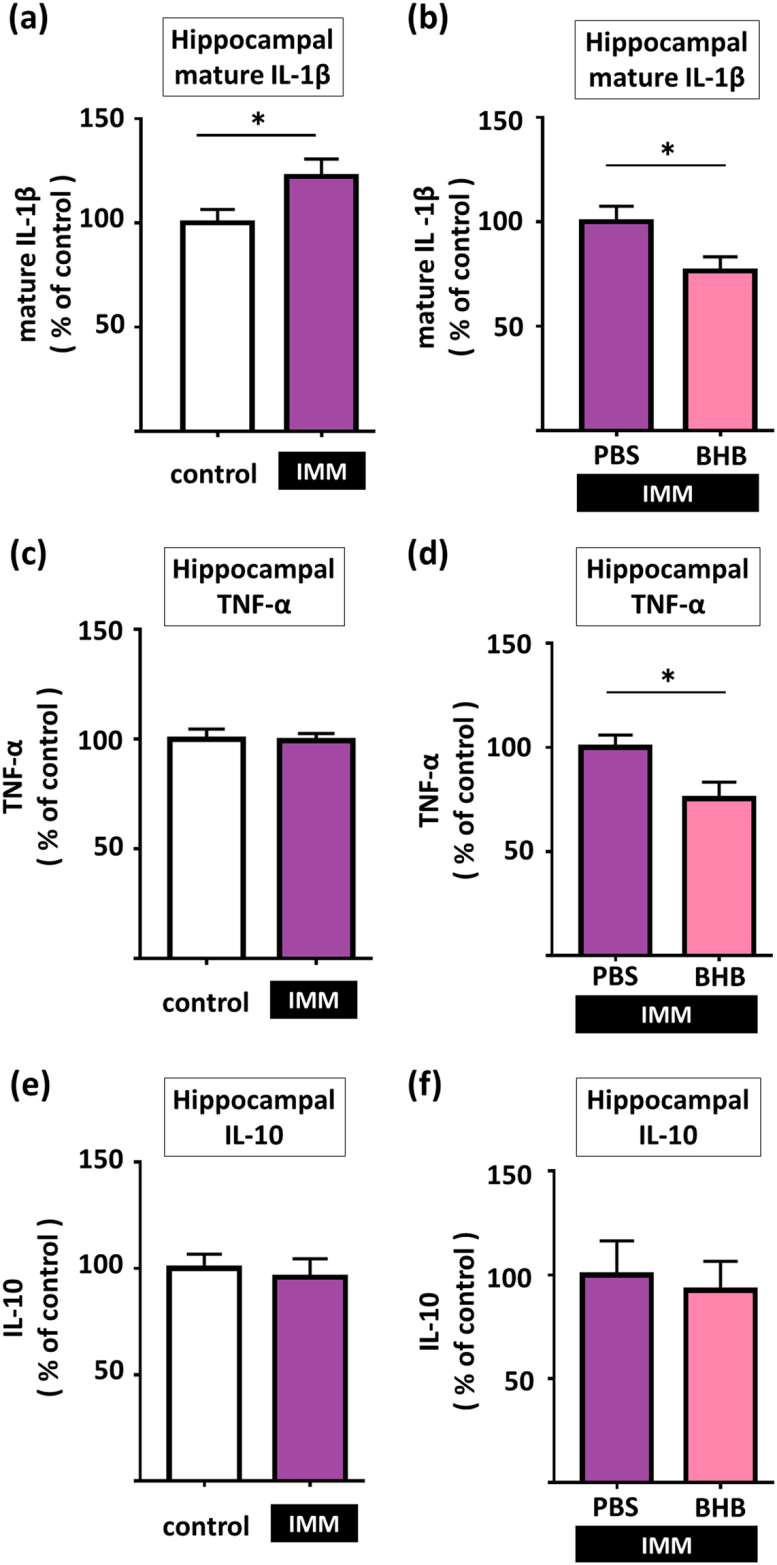
Pretreatment with BHB reduces stress-induced increases in pro-inflammatory cytokine levels in the hippocampus. (**a**) IL-1β levels are shown. One hour of IMM stress increased levels of IL-1β in the hippocampus. (**b**) IL-1β levels are shown. BHB pretreatment decreased levels of IL-1β in the hippocampus in IMM model rats. (**c**) Hippocampal TNF-α levels after 1 h IMM stress are shown. 1 h of IMM did not change TNF-α levels in the hippocampus. (**d**) Hippocampal TNF-α levels after 1 h of IMM with pretreatment by BHB are shown. BHB pretreatment decreased TNF-α levels in the hippocampus in IMM model rats. (**e**) Hippocampal IL-10 levels after 1 h IMM stress are shown. 1 h of IMM did not change IL-10 levels in the hippocampus. (**f**) Hippocampal IL-10 levels after 1 h of IMM with pretreatment by BHB are shown. BHB pretreatment did not change IL-10 levels in the hippocampus in IMM model rats. IMM: immobilization stress, PBS: phosphate buffered saline, BHB: beta-hydroxybutyrate, *p < 0.05, Error bars, standard error of the mean (SEM).

We measured levels of IL-1β protein (17 kDa) and TNF-α (30 kDa) as well as interleukin-10 (IL-10) (18 kDa) in the hippocampus after acute IMM stress or CUS via western blotting. The levels of IL-1β were significantly increased in rats exposed to IMM stress as compared to control non-stressed rats (t43 = 2.126, p = 0.039, n = 22–23) (Fig. 3a). A single dose of subcutaneously administered BHB reduced the hippocampal IL-1β levels in IMM-stressed rats (t12 = 2.360, p = 0.036, n = 7) (Fig. 3b). We found no change in TNF-α levels in the hippocampus at 1 h after IMM (t 43 = 0.154, p = 0.878, n = 22–23) (Fig. 3c). However, subcutaneous administration of a single dose of BHB significantly reduced hippocampal TNF-α levels at 1 h after IMM (t12 = 2.487, p = 0.029, n = 7) (Fig. 3d). IMM did not change the levels of IL-10 (t43 = 0.375, p = 0.709, n = 22–23) (Fig. 3e); moreover, BHB administration did not alter the levels of IL-10 (t10 = 0.323, p = 0.753, n = 5–7) (Fig. 3f). Neither IMM nor BHB administration altered the levels of β-actin in the hippocampus (data not shown).

We also evaluated the levels of cytokines (IL-1β, TNF-α, and IL-10) in the hippocampus after BHB administration, without any acute stress (without an immobilization stress). The result showed that BHB alone did not result in any change in cytokine levels relative to the control group (PBS) (Supplementary Figure 1g–i).

**Figure 4:**
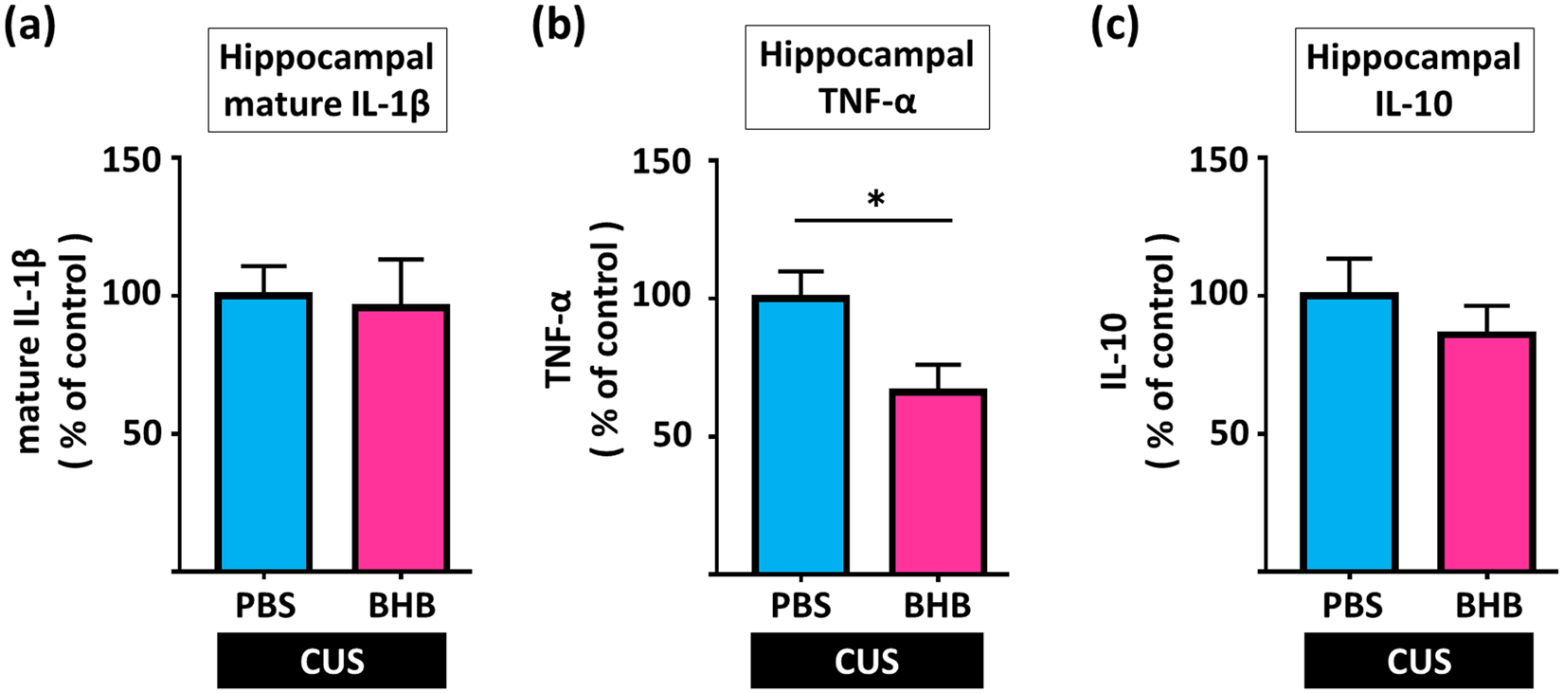
Chronic peripheral BHB treatment reduces levels of TNF-α, but not of IL-1β, in the hippocampus after CUS. (**a**) IL-1β levels in the hippocampus are shown. Chronic BHB treatment did not alter levels of IL-1β in the hippocampus in CUS model rats. (**b**) Hippocampal TNF-α levels in the hippocampus are shown. Chronic BHB treatment decreased levels of TNF-α in the hippocampus in CUS rats. (**c**) IL-10 levels in the hippocampus are shown. Chronic BHB treatment did not alter levels of IL-10 in the hippocampus in CUS model rats. CUS: chronic unpredictable stress, PBS: phosphate buffered saline, BHB: beta-hydroxybutyrate, *p < 0.05, Error bars, standard error of the mean (SEM).

Next, we measured the levels of IL-1β, TNF-α and IL-10 in the hippocampus after CUS. Although chronic BHB administration did not alter the levels of IL-1β (t15 = 0.205, p = 0.840, n = 8–9) and IL-10 (t15 = 0.831, p = 0.419, n = 8–9) after 6 weeks of CUS (Fig. 4a and c), chronic BHB administration significantly decreased levels of TNF-α in the hippocampus after CUS (t 15 = 2.396, p = 0.030, n = 8–9) (Fig. 4b). Chronic BHB administration did not alter levels of β-actin in the hippocampus (data not shown).

**Figure 5:**
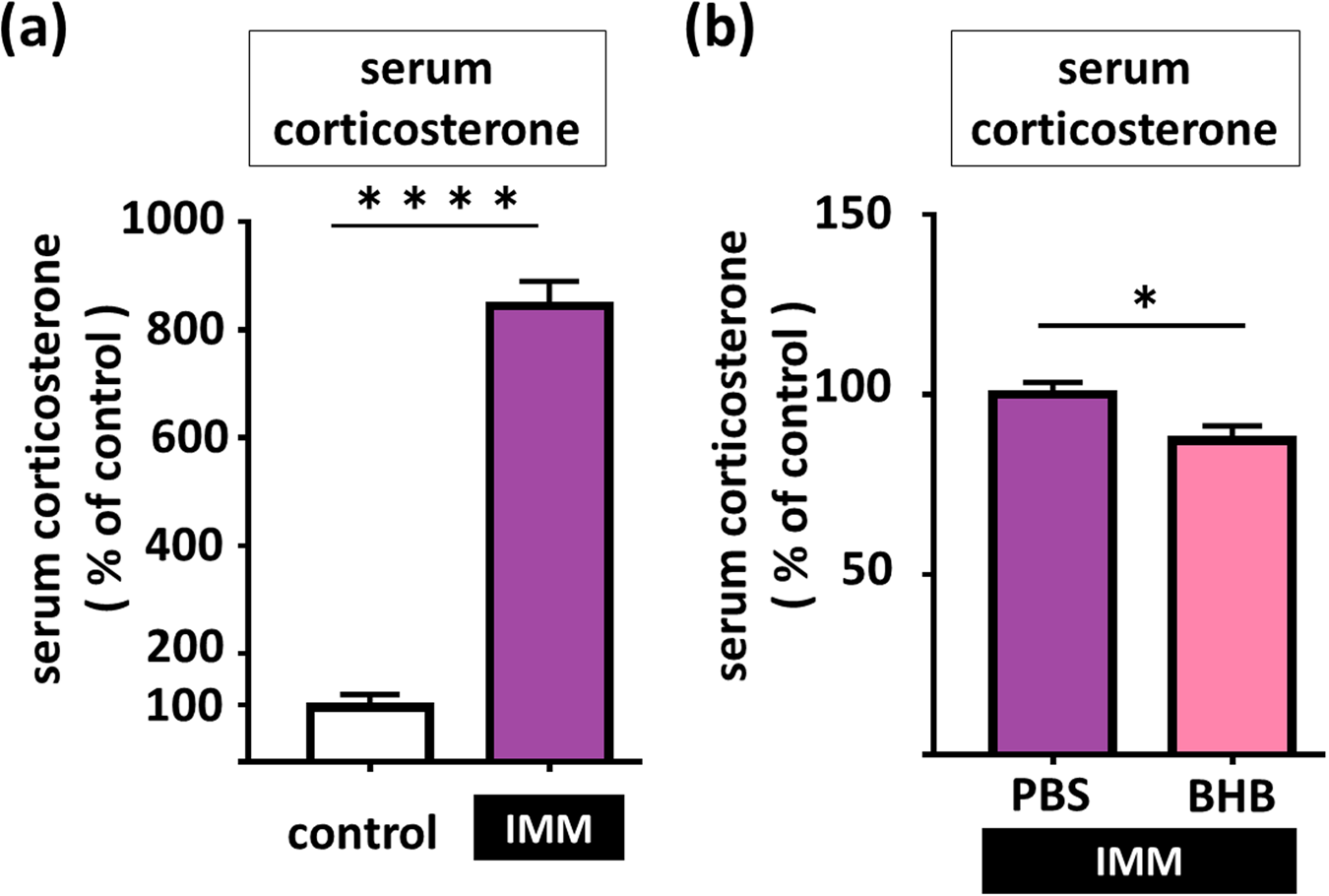
Pretreatment with BHB attenuates stress induced-serum corticosterone levels in IMM stress rats. (**a**) Serum corticosterone levels at 1 h after IMM is shown. 1 h of IMM significantly increased serum corticosterone levels. (**b**) Serum corticosterone levels at 1 h after IMM stress, with pretreatment by BHB are shown. BHB pretreatment decreased serum corticosterone level in IMM model rats. IMM: immobilization stress, PBS: phosphate-buffered saline, BHB: beta hydroxybutyrate, *p < 0.05, ****p < 0.0001, Error bars, standard error of the mean (SEM).

We also found that exposure to 1 h of IMM stress markedly elevated serum corticosterone levels (t8 = 14.37, p < 0.0001, n = 5) (Fig. 5a), but pretreatment with BHB significantly reduced this effect (t12 = 2.380, p = 0.035, n = 7) (Fig. 5b). The result showed that BHB alone did not change serum corticosterone level relative to the control group (PBS) (Supplementary Figure 1j).

## Discussion

In the present study, we observed that BHB administration produced antidepressant-like effects in a rat model of CUS-induced depression. We also examined the influence of BHB on hippocampal inflammation induced by acute or chronic stress, and found that BHB attenuated stress-induced increases in IL-1β and TNF-α in the hippocampus. The present study provided new evidence that BHB exerts antidepressant-like effects likely by inhibiting stress-induced increases in the levels of IL-1β and TNF-α.

BHB is a ketone body synthesized in the liver of mammals, which serves as an alternative energy source for the brain during physiological states characterized by limited carbohydrate and surplus fatty acid availability^13, 14^. Some basic research has suggested that BHB may have a beneficial effect in various neurodegenerative conditions, such as Huntington’s disease, Parkinson’s disease, and Alzheimer’s disease, by preventing striatal histone deacetylation, improving mitochondrial respiration, and inhibiting amyloid-β aggregation and brain atrophy^16–18^. Furthermore, intravenous administration of BHB protects the rat brain against damage caused by focal cerebral ischemia, possibly by decreasing oxygen radical production^19^. These lines of evidence suggest that BHB may exert neuroprotective effects in several diseases of the central nervous system.

Moreover, basic research studies have reported that BHB displays anti-inflammatory effects via inhibition of the NLRP3 inflammasome. BHB suppresses lipopolysaccharide-induced IL-1β elevation in BV-2 cells by inhibiting nuclear factor-kappa B activation^20^. BHB also suppresses activation of the NLRP3 inflammasome and reduces NLRP3 inflammasome-mediated IL-1β and interleukin 18 (IL-18) production in human monocytes and in mouse models of NLRP3-mediated diseases, such as Muckle–Wells syndrome^15^. BHB deactivates the neutrophil NLRP3 inflammasome, relieving gout flares^21^. Qian *et al*. reported that BHB suppresses NLRP3 inflammasome activation and promotes functional recovery in mice with spinal cord injury^22^. These findings indicate that BHB inhibits activation of the NLRP3 inflammasome, resulting in suppression of IL-1β during cerebral infection, ischemia, and psychological stress.

As some research has indicated that peripheral BHB crosses the blood–brain barrier, we first examined whether peripherally administered BHB could indeed enter the brain, and determined the optimum injection timing of BHB for the subsequent experiments (Fig. 1)^23^. BHB levels increased in the hippocampus at 1 h after BHB administration, in accordance with the findings of a previous report on the effect of intragastric administration of a BHB methyl ester^18^, although the increase in BHB in the present study was comparatively smaller. This difference in BHB levels may be due to differences in the route or dose of administration. In the present study, although the extent of the BHB increase was small, our results indicated that peripherally administered BHB indeed enters the brain.

We then evaluated the antidepressant-like effects of BHB using a CUS procedure similar to that used in our previous studies^8, 24^. The CUS paradigm is an animal model of depression with high face, construct, and predictive validity^25–27^. We confirmed that rats exposed to CUS exhibited depressive behavioral changes (Fig. 2b–f). These behavioral changes are considered to be evidence of despair, anhedonia, and anxiety in rodents^28–31^. Moreover, our results indicate that chronic BHB administration prevents the development of these CUS-induced behaviors, which is regarded as a characteristic feature of antidepressant drugs (Fig. 2b–f)^32^. Although there have been no previous reports indicating that BHB produces antidepressant-like effects, there is some evidence suggesting that a ketogenic diet – which elevates levels of BHB in the brain – may be associated with antidepressant effects. For example, it has been reported that a ketogenic diet decreases the time spent immobile in the FST^33^, while another report demonstrated that adult offspring exposed to a prenatal ketogenic diet exhibited reduced susceptibility to anxiety and depression^34^.

We also found that acute stress elevates IL-1β levels in the hippocampus (Fig. 3a), consistent with our previous report^8^, and that peripheral BHB administration attenuates this effect (Fig. 3b). Our previous findings had indicated that stress activates NLRP3 and releases IL-1β and TNF-α, and injection of BHB, which is an endogenic NLRP3 inhibitor, is thought to ameliorate the production of these proinflammatory cytokines^8^. IL-1β is a pro-inflammatory cytokine and a key mediator of a variety of behavioral actions associated with stress^9^. Moreover, accumulating evidence has suggested that inhibition of IL-1β exerts antidepressant-like effects. Infusion of IL-1β decreases neurogenesis in the adult hippocampus and causes depressive behavior, while administration of an IL-1β receptor antagonist blocks CUS-induced decreases in neurogenesis and reduces anhedonic behavior^5^. Furthermore, IL-1 receptor-null mutant mice exhibit decreased anhedonic and anxiety-like behavior^5, 7^. Additionally, some clinical studies have suggested that pro-inflammatory cytokines play a crucial role in the pathophysiology of MDD^35^. Serum levels of pro-inflammatory cytokines, including IL-1β, are increased in patients with MDD^3, 4^, and these effects are normalized after antidepressant treatment^36^. Increased levels of IL-1β have also been observed in the cerebrospinal fluid of patients with MDD^37^. Although blockade of IL-1β is a viable therapeutic target for the treatment of depression, this approach faces several problems, such as the risk of serious infections due to systemic inhibition of IL-1β^9^.

The NLRP3 inflammasome is composed of NLRP3, adapter protein ASC, and pro-caspase-1. NLRP3 is a member of the NLR family of cytosolic pattern-recognition receptors. Resident microglia express NLRP3 in the central nervous system^38^. When NLRP3 detects various danger signals, it is activated to form the inflammasome, which cleaves pro-caspase-1 to mature caspase-1, which in turn cleaves pro-IL-1β to mature IL-1β^10^. One clinical study has reported increased gene expression of NLRP3 in mononuclear blood cells and increased levels of serum IL-1β and IL-18 in patients with pharmacologically untreated MDD. Interestingly, both of these effects were reversed by treatment with amitriptyline^39^. These results indicate an association between depression and levels of pro-inflammatory cytokines via NLRP3.

Recently, we demonstrated that psychological stress elevates extracellular adenosine triphosphate (ATP), IL-1β, and TNF-α levels, and activates the NLRP3 inflammasome in the adult hippocampus^8^. The P2X7 R, an ATP receptor on microglia, is required for activation of the inflammasome and processing of IL-1β. Pretreatment with a P2X7 R antagonist inhibited these changes, and chronic administration of a P2X7 R antagonist exerted an antidepressant effect. These results indicate that stress-induced P2X7 R stimulation increases levels of the active form of the NLRP3 inflammasome complex, which cleaves and releases IL-1β, and subsequently releases TNF-α, resulting in the development of depression^8^. Based on this evidence, we propose that NLRP3 inflammasome activation plays a critical role in stress-induced neuro-inflammation and depression, and that inhibition of the NLRP3 inflammasome may have antidepressant effects^11, 12^. Our present study supports this hypothesis and suggests that BHB, which is an endogenous NLRP3 inflammasome inhibitor, may exert antidepressant effects via inhibition of the NLRP3 inflammasome under conditions of stress.

Furthermore, we found that both single-dose and chronic BHB treatment reduced TNF-α levels in the hippocampus (Figs 3 d and 4b). TNF-α is not directly regulated by the NLRP3 inflammasome. However, our previous study revealed that acute stress increases hippocampal TNF-α levels with a brief time lag after elevation of IL-1β, and pretreatment with a P2X7 R antagonist blocks these responses, suggesting that IL-1β stimulates the release of TNF-α^8^. TNF-α is known to have an anti-neurogenic effect and to induce depression-like behavior^40^. Together, these findings suggest that BHB attenuates TNF-α levels indirectly through inhibition of the IL-1β cytokine cascade, and that this could contribute to the antidepressant actions of BHB.

BHB failed to reduce IL-1β levels after the CUS exposure (Fig. 4a). This result is consistent with those of our previous study, which showed that IL-1β increases only immediately after exposure to a stressor, and then immediately returns to basal levels; thus we may not be able to detect IL-1β changes in the absence of acute stress^8^. On the other hand, we observed a decrease in TNF-α levels after treatment with BHB in the CUS group (Fig. 4b). This may also be consistent with the findings of our previous study that showed that the increase of TNF-α lasts longer, so that changes in TNF-α levels remain detectable for a longer period^8^. A meta-analysis of clinical studies reporting that TNF-α levels are consistently increased in MDD patients^4^ is consistent with our findings of a reduction of TNF-α levels in CUS rats that had been treated with BHB.

We also demonstrated that BHB partly attenuates the IMM stress-induced increase of serum corticosterone levels (Fig. 5a and b). IL-1β increases the release of hypothalamic corticotropin-releasing hormone (CRH), secretion of pituitary adenocorticotropic hormone (ACTH), and adrenal steroidogenesis^11, 41–43^. Sustained levels of glucocorticoids can cause atrophy of the pyramidal neurons in the hippocampus, and thereby contribute to depressive symptoms^11, 44^. Thus, it is possible that a decrease in the stress-induced levels of corticosterone contributes to attenuation of stress-induced depressive behaviors as observed in the present study.

There are some limitations in the present study. Although we demonstrated the antidepressant-like effects of BHB, the treatment paradigm used shows a preventative action, rather than reversal of the CUS effects. Further studies are required to assess the therapeutic efficacy of BHB in animal models of depression. In addition, we did not evaluate how BHB affects the NLRP3 inflammasome under conditions of stress. Therefore, it remains unclear whether BHB reduces stress-induced increases in IL-1β via inhibition of NLRP3. Furthermore, although the behaviors of rats exposed to CUS tended toward depression, we failed to demonstrate statistically significant depressive behavior in some tests in the CUS model, even though we used similar stressors as in previous studies^27^, and a relatively large number of animals per group (n = 16). It is also worth noting that the extent of BHB increase in the brains of animals in the present study was small in comparison to that reported in a previous neuroprotective study in a mouse model of Alzheimer’s disease^18^. Nevertheless, our findings successfully demonstrated the anti-inflammatory effect of BHB after subcutaneous administration of a single dose. Also, because the levels of IL-1β and TNF-α in the hippocampus were significantly suppressed by BHB, that small increase appears to be physiologically relevant. Thus, it is possible that a single dose of BHB may produce a slight increase in brain levels of BHB, which may be sufficient to improve symptoms of depression. However, further studies are required to elucidate the mechanisms underlying the antidepressant and anti-inflammatory effects of BHB in mouse models of depression.

## Conclusion

The findings of the present study demonstrate that BHB treatment exerts antidepressant-like effects in a CUS-induced rat model of depression, and that BHB attenuates stress-induced increases in hippocampal levels of IL-1β. As IL-1β, which is thought to be tightly regulated by NLRP3 inflammasome, plays a critical role in stress-induced neuro-inflammation and in the development of depressive states, BHB may exert its antidepressant-like effects via blockade of NLRP3-induced increases in hippocampal levels of IL-1β. BHB is an endogenic NLRP3 inflammasome inhibitor, which is thought to be a safe and versatile physiologically active substance and therefore has some advantages for clinical applications. These results are critical for the development of new pharmacotherapeutic approaches that target neuro-inflammation in MDD.

## Method

### Animals and housing

Male Sprague–Dawley (SD) rats with initial weights of 310–370 g (9–10-weeks-old) were obtained from Charles River Laboratories (Yokohama, Japan). Animals were housed three rats per cage under a 12-h light/dark cycle (lights on from 7:30 am to 7:30 pm) at a constant temperature (25 °C), with free access to food and water, except during exposure to CUS, and were allowed to acclimate to the housing environment for 7 days. Behavioral testing was conducted between 10:00 am and 4:00 pm, with the exception of the sucrose preference test. All experimental procedures involving animals were conducted in accordance with the Institutional Animal Care Guidelines and approved ethically by the Tottori University Animal Care and Use Committee (approval number 14-Y-33). All efforts were made to minimize animal suffering.

### Experimental design

**Figure 6:**
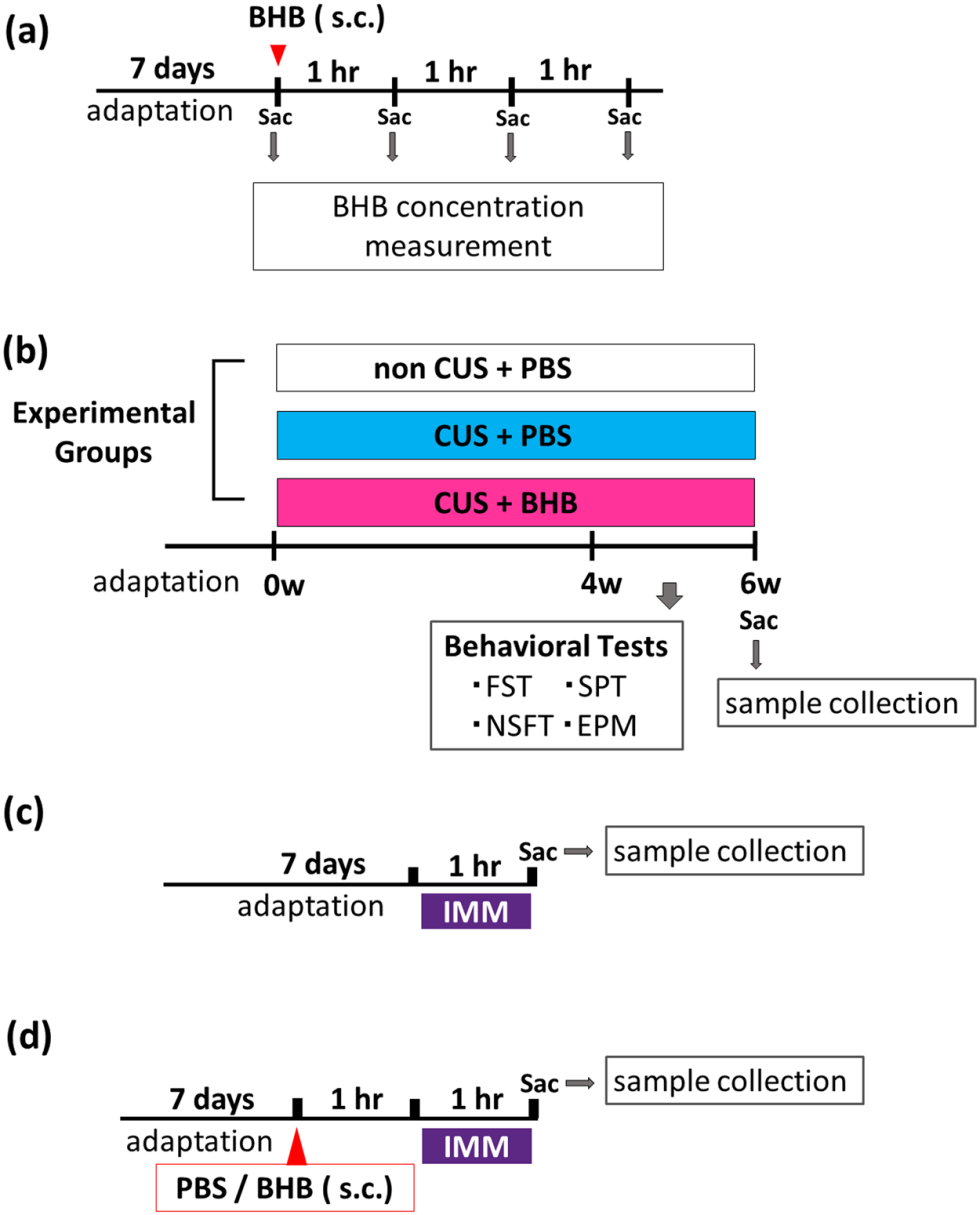
Experimental procedures. (**a**) The procedure for measurement of hippocampal BHB concentration is shown. In each group, four rats were treated with 250 mg/kg BHB subcutaneously, and were then sacrificed for sample collection at 1, 2, and 3 h after BHB injection. (**b**) Schematic representation of the CUS experimental procedure and behavioral tests. Three groups were studied: non-CUS + PBS, CUS + PBS, and CUS + BHB, n = 16 per group. Rats were exposed to two variable sequences of unpredictable stressors per day in the daytime and night. All rats were administered PBS or BHB (250 mg/kg) subcutaneously, twice a day. Behavioral tests were conducted after 4 weeks of CUS. (**c**) The experimental paradigm for IMM stress is shown. Rats were sacrificed after exposure to 1 h of stress, and the brain and blood were collected. (**d**) The experimental paradigm for pretreatment and IMM stress is shown. PBS or BHB was subcutaneously administered 1 h prior to IMM, and rats were sacrificed after 1 h of IMM stress, and the brain and blood were collected. CUS: chronic unpredictable stress, PBS: phosphate buffered saline, BHB: beta-hydroxybutyrate, IMM: immobilization stress, Sac: sacrifice, s.c.: subcutaneous injection, FST: forced swim test, SPT: sucrose preference test, NSFT: novelty suppressed feeding test, EPM: elevated plus maze test.

Experimental procedures are described in Fig. 6. To determine the optimum timing for BHB injection, 16 rats (four rats in each group) were used for the measurement of hippocampal BHB concentrations. BHB was subcutaneously administered at a dosage of 250 mg/kg. Rats were then sacrificed for sample collection at 0 (pre-injection), 1, 2, and 3 h after BHB injection (Fig. 6a). Next, to evaluate the effect of BHB for the animal model of depression, rats were divided into three groups of 16 each for the chronic stress experiment, as follows: non CUS + phosphate-buffered saline (PBS), CUS + PBS, and CUS + BHB (PBS or BHB was injected subcutaneously just before each stressor, twice a day). After 4 weeks of CUS, behavioral tests were performed while continuing CUS (Fig. 6b). After a total of 6 weeks of CUS, rats were sacrificed and the levels of IL-1β, TNF-α, and IL-10 were measured. Tissue collection was done 12 h after administration of the last stressor of the CUS paradigm was completed. To elucidate the effects of acute stress (IMM) on pro-inflammatory cytokine expression, rats were sacrificed after 1 h of IMM and the levels of cytokines and corticosterone were measured (Fig. 6c). Additionally, to evaluate the anti-inflammatory effects of BHB, PBS or BHB were injected 1 h prior to IMM. Rats were sacrificed after 1 h of IMM stress and the cytokine and corticosterone levels were measured (Fig. 6d).

### Drugs and reagents

DL-BHB (Tokyo Chemical Industry, Tokyo, Japan) was dissolved in PBS (NaCl 137 mM, KCl 2.7 mM, Na_2_HPO_4_·12H_2_O 8.1 mM, KH_2_PO_4_ 1.47 mM, pH 7.5) to a concentration of 150 mg/mL and adjusted to a pH of 7.5.

### Measurement of hippocampal BHB concentration

The brains were rinsed with PBS to remove any red blood cells or clots. Rat hippocampus was dissected on ice, and the samples were immediately frozen at −80 °C until required for use. Tissue samples were homogenized with β-HB Assay Buffer (Item No. 700191, Cayman Co Ltd, Ann Arbor, MI, USA) and centrifuged at 10,000 × *g* to obtain the supernatant. The concentrations of BHB in the supernatant were assayed using the BHB assay kit (Item No. 700190, Cayman Co Ltd) according to the manufacturer’s protocol.

### Chronic unpredictable stress procedure

We utilized a CUS procedure to create a rodent model of depression. Rats were exposed to two variable sequences of unpredictable stressors per day, both in the daytime and at night. The stressors included cage tilt (45°, overnight), lights off during the daytime, lights on overnight, crowding (eight rats per cage, overnight), cold stress (20 min, daytime), small cages (cage for mice, overnight or during the daytime), cage rotation (1 h, daytime), immobilization stress (1 h, daytime), wet bedding (overnight or during the daytime), isolation (overnight), and food and water deprivation (24 h), followed by empty water bottle replacement (4 h). These stressors were randomly scheduled throughout the 4-week experiment. Control rats were handled every day without CUS.

### Immobilization Stress

We utilized the IMM method to induce acute stress. Rats were restrained using conical plastic film tubes for 1 h.

### Behavioral testing

#### Forced swim test

A single session of the FST was conducted as previously described^45^. Rats were forced to swim individually in a plastic bucket (32 cm × 47 cm) filled with water (24 °C, 34 cm depth) for 6 min. Tests were recorded on videotape and later scored by an observer who was blinded to the animal groups. The cumulative time spent in immobile posturing (floating in the water with only movements necessary to keep the nose above the surface) was recorded. The water was changed after each trial.

#### Sucrose preference test

Animals were acclimated to 1% sucrose for 48 h (Wako Pure Chemical Industries Ltd., Osaka, Japan). One week later, preference for 1% sucrose or water was determined over an 8-h period (overnight). The preference for sucrose was calculated as (mL sucrose/mL total consumed) × 100.

#### Novelty-suppressed feeding test

The NSFT was conducted as previously described^8^. After 24 h of food deprivation, the NSFT was performed in an open field (90 cm × 90 cm × 45 cm), and the latency to feed was recorded, up to a maximum of 20 min. The open field was cleaned after each trial, and all tests were conducted under dimly lit conditions.

#### Elevated plus maze test

The elevated plus maze consisted of two open arms (50 cm × 10 cm) and two closed arms (50 cm × 10 cm; 30-cm high walls) arranged such that the two open arms were opposite each other. The maze was elevated to a height of 50 cm. Rats were gently placed in the closed arm and were allowed to explore the maze for 5 min. The total time spent in each arm as well as the number of entries into each arm was recorded. The maze was cleaned after each trial. The test was conducted under dimly lit conditions.

### Measurement of IL-1β in the hippocampus

We measured hippocampal levels of IL-1β by western blotting. The frozen hippocampal tissue samples were homogenized in ice-cold buffer (320 mM sucrose, 20 mM HEPES, 1 mM EDTA, 1.25 mM NaF, and 1 mM NaVO3) containing protease inhibitor (cOmplete® Cocktail tablets, Roche Diagnostics GmbH, Mannheim, Germany). The samples were then centrifuged at 500 × *g* for 1 min at 4 °C to remove debris, and supernatant was collected. Supernatant was centrifuged at 12,000 × *g* for 10 minutes at 4 °C, after which the supernatant was again collected. Total protein concentrations were determined using the bicinchoninic acid (BCA) assay kit (Pierce® BCA Protein Assay Kit, Thermo Scientific, Rockford, IL, USA).

Protein samples were separated using 12% sodium dodecyl sulfate-polyacrylamide gel electrophoresis and transferred to nitrocellulose membranes. Nonspecific protein-binding sites were blocked with Tris-buffered saline containing 0.1% Tween-20 (TBST, Tris-HCl 20 mM, NaCl 102 mM, pH 7.0) and 1% bovine serum albumin for 30 min, at room temperature (20–25 °C), and incubated with primary antibodies for IL-1β (ab9722; 1:2500 dilution, i.e., 0.2 μg/mL, Abcam, Cambridge, MA, USA) diluted into TBST at 4 °C overnight. After 20 min of washing with TBST (three times), membranes were incubated with a secondary antibody to rabbit (ab16284; 1:10000, i.e., 0.1 μg/mL, Abcam, Cambridge, MA, USA) diluted into TBST at room temperature for 1 h. After 20 min of washing (three times), western blot bands were visualized by incubation with a chemiluminescent substrate (ECL Select Western Blotting Detection Reagent, GE Healthcare, Little Chalfont, UK), and the membranes were scanned using an imager (AE-6981 Light CaptureII 130W, ATTO CORPORATION, Tokyo, Japan). Densitometric analysis of western blot bands was performed using software ImageJ Version 1.51 (National Institutes of Health, Bethesda, MD, USA). Membranes were stripped and re-probed with primary antibodies against β-actin (A1978; 1:4000 dilution; i.e., 0.5 μg/mL, Sigma–Aldrich, St. Louis, MO, USA) and secondary antibody for mouse (ab97023; 1:10000 dilution; i.e., 0.1 μg/mL; Abcam, Cambridge, MA, USA).

### Measurement of TNF-α and IL-10 in the hippocampus

Hippocampal TNF-α and IL-10 levels were measured via western blotting. Western blotting was performed as mentioned above, but without a blocking step. Primary antibodies for TNF-α (AB1837P; 1:2500, i.e., 0.2 μg/mL, Merck Millipore, Darmstadt, Germany) and for IL-10 (ab9969; 1:10000 dilution; i.e., 0.1 μg/mL; Abcam, Cambridge, MA, USA), and secondary antibody for rabbit (ab16284 1:10000 = 0.1 μg/mL, Abcam, Cambridge, MA, USA) were used.

### Serum corticosterone measurement

The blood was collected after 1 h of acute immobilization stress. The blood was stored at 4 °C overnight. The supernatant was collected, and then the serum corticosterone level was measured by the CLEIA method, in accordance with the manufacturer’s instructions (Roche Diagnostics K.K, Tokyo, Japan).

### Statistical analyses

All statistical analyses were conducted using SPSS Statistics 19.0 (Tokyo, Japan). Data were analyzed using unpaired, two-tailed *t*-tests. For comparisons among the three groups, analyses of variance were conducted, followed by Tukey’s *post-hoc* test. Some behavioral data (SPT, NSFT, time spent in open arms in the EPM, and number of entries into the open arms in the EPM) were not normally distributed based on the Shapiro–Wilk test. Therefore, we used the Kruskal–Wallis test and Mann–Whitney U test for comparisons between those groups. As previously described^8^, values were excluded from analysis if they were greater than two standard deviations (SD) from the mean. The data are presented as mean ± standard error of the mean (SEM). p-values < 0.05 were considered significant.

## Electronic supplementary material

Supplementary Figure 1
