# Supplementary material for: Beta-hydroxybutyrate, an endogenic NLRP3 inflammasome inhibitor, attenuates stress-induced behavioral and inflammatory responses
Source: Sci Rep. 2017 Aug 9;7:7677. doi: 10.1038/s41598-017-08055-1 (PMC5550422; doi:10.1038/s41598-017-08055-1)
Supplement: Supplementary file 1 — Supplementary Figure 1 [file 41598_2017_8055_MOESM1_ESM.pdf]

Beta-hydroxybutyrate, an endogenic NLRP3 inflammasome inhibitor, attenuates stress-induced behavioral and inflammatory responses

Takehiko Yamanashi M.D.[1], Masaaki Iwata M.D., Ph.D.[1]*, Naho Kamiya[1], Kyohei Tsunetomi[1], Naofumi Kajitani M.D.[1], Nodoka Wada[1], Takahiro Iitsuka[1], Takahira Yamauchi M.D., Ph.D.[2], Akihiko Miura M.D.[1], Shenghong Pu Ph.D.[1], Yukihiko Shirayama M.D., Ph.D.[3], Ken Watanabe M.D., Ph.D.[4], Ronald S. Duman Ph.D.[5], and Koichi Kaneko M.D., Ph.D.[1]

[1]Department of Neuropsychiatry, Faculty of Medicine, Tottori University, Yonago, Japan
[2]Department of Psychiatry, Nara Medical University School of Medicine, Kashihara, Japan
[3]Department of Psychiatry, Teikyo University Chiba Medical Center, Ichihara, Japan
[4]Watanabe Hospital, Tottori, Japan
[5]Departments of Psychiatry and Neurobiology, Yale University School of Medicine, New Haven, Connecticut, USA

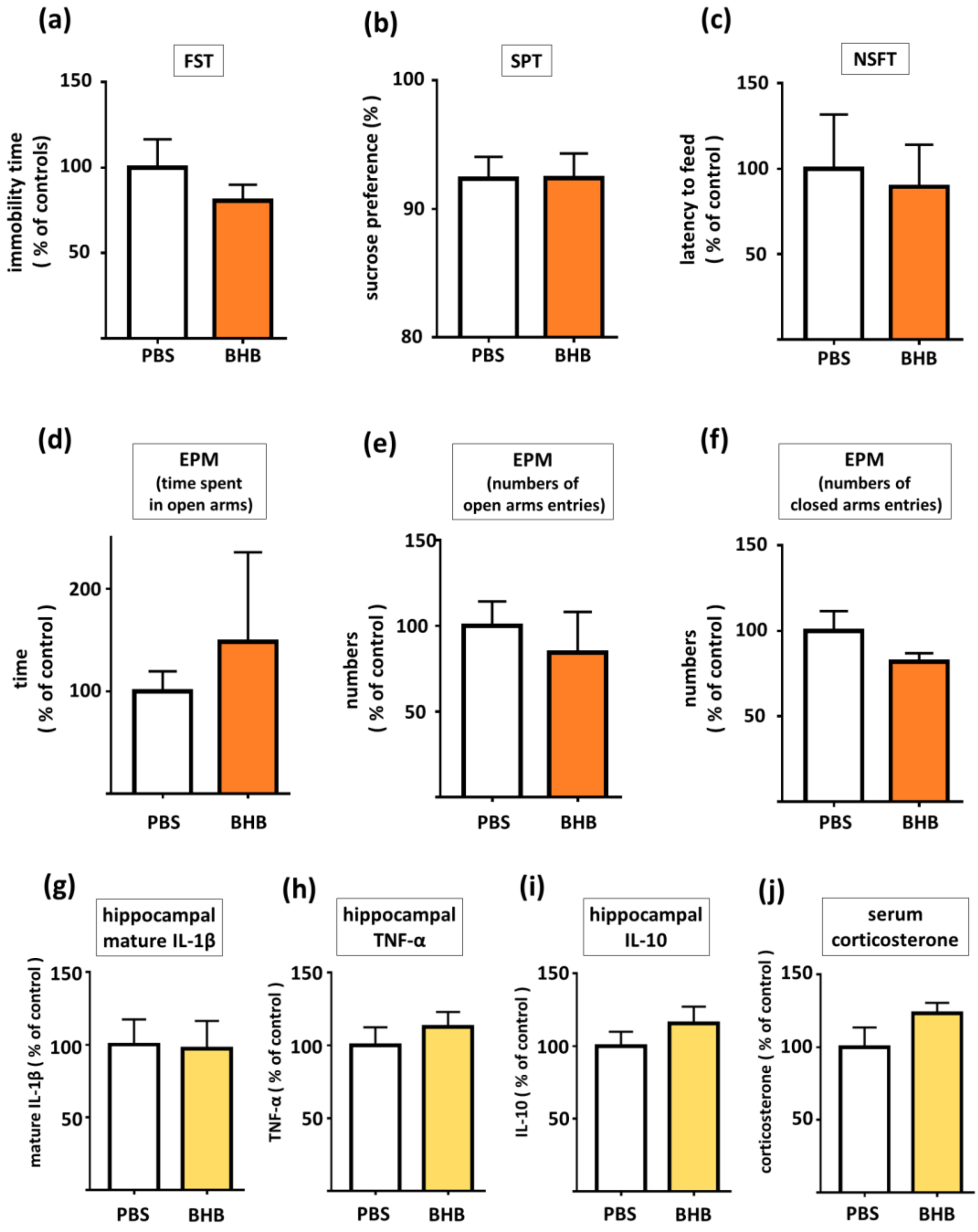

Supplementary Figure 1. BHB alone does not change in behavior or cytokine levels. 3 weeks BHB administration without the CUS paradigm did not change or improve behaviors in (a) FST, (b) SPT, (c) NSFT, (d) time spent in open arms in the EPM, (e) numbers of open arms entries in the EPM, and (f) numbers of closed arms entries in the EPM. Additionally, single administration of BHB without any acute stress did not change the levels of cytokines such as (g) IL-1β, (h) TNF-α, and (i) IL-10, also (j) the levels of serum corticosterone.
